# Pulmonary Mesenchymal Stem Cells in Mild Cases of COVID-19 Are Dedicated to Proliferation; In Severe Cases, They Control Inflammation, Make Cell Dispersion, and Tissue Regeneration

**DOI:** 10.3389/fimmu.2021.780900

**Published:** 2022-01-13

**Authors:** Andrea Henriques-Pons, Daniela Gois Beghini, Vanessa dos Santos Silva, Samuel Iwao Horita, Fabrício Alves Barbosa da Silva

**Affiliations:** ^1^ Fundação Oswaldo Cruz, Instituto Oswaldo Cruz, Laboratório de Inovações em Terapias, Ensino e Bioprodutos, Rio de Janeiro, Brazil; ^2^ Fundação Oswaldo Cruz, Programa de Computação Científica, Rio de Janeiro, Brazil

**Keywords:** mesenchymal stem cell, COVID-19, cell therapy, single cell RNA sequencing, cytokine storm

## Abstract

Mesenchymal stem cells (MSCs) are multipotent adult stem cells present in virtually all tissues; they have potent self-renewal capacity and differentiate into multiple cell types. For many reasons, these cells are a promising therapeutic alternative to treat patients with severe COVID-19 and pulmonary post-COVID sequelae. These cells are not only essential for tissue regeneration; they can also alter the pulmonary environment through the paracrine secretion of several mediators. They can control or promote inflammation, induce other stem cells differentiation, restrain the virus load, and much more. In this work, we performed single-cell RNA-seq data analysis of MSCs in bronchoalveolar lavage samples from control individuals and COVID-19 patients with mild and severe clinical conditions. When we compared samples from mild cases with control individuals, most genes transcriptionally upregulated in COVID-19 were involved in cell proliferation. However, a new set of genes with distinct biological functions was upregulated when we compared severely affected with mild COVID-19 patients. In this analysis, the cells upregulated genes related to cell dispersion/migration and induced the γ-activated sequence (GAS) genes, probably triggered by IFNGR1 and IFNGR2. Then, *IRF-1* was upregulated, one of the GAS target genes, leading to the interferon-stimulated response (ISR) and the overexpression of many signature target genes. The MSCs also upregulated genes involved in the mesenchymal-epithelial transition, virus control, cell chemotaxis, and used the cytoplasmic RNA danger sensors RIG-1, MDA5, and PKR. In a non-comparative analysis, we observed that MSCs from severe cases do not express many NF-κB upstream receptors, such as Toll-like (TLRs) TLR-3, -7, and -8; tumor necrosis factor (TNFR1 or TNFR2), RANK, CD40, and IL-1R1. Indeed, many NF-κB inhibitors were upregulated, including *PPP2CB*, *OPTN*, *NFKBIA*, and *FHL2*, suggesting that MSCs do not play a role in the “cytokine storm” observed. Therefore, lung MSCs in COVID-19 sense immune danger and act protectively in concert with the pulmonary environment, confirming their therapeutic potential in cell-based therapy for COVID-19. The transcription of MSCs senescence markers is discussed.

## Introduction

Mesenchymal stem cells **(**MSCs) were described by Friedenstein in 1970 (1) and were first isolated from the bone marrow as non-hematopoietic stem cells. They are undifferentiated adherent spindle-shaped cells found in virtually all adult tissues and facilitate tissue remodeling and repair throughout adult life (2). Considering that MSCs would differentiate only into mesodermal cells, such as bone, cartilage, tendon, and fat, in 1991 Caplan introduced the term “mesenchymal stem cells” (3–5). However, today we know that MSCs are pluripotent stem cells, as they can also differentiate into ectodermal (6) and endodermal (7) cell lineages. The identification of human MSCs is based on their capacity to adhere to plastic and on markers expressed by *in vitro* expanded cells (8), with the canonical phenotype of CD73^+^CD90^+^CD105^+^ cells and no expression of CD34, CD45, CD14, CD11B, and CD3ϵ. Moreover, they must differentiate into three cell lineages, adipocytes, chondrocytes, and osteocytes, under inductive culture conditions (8). MSCs also seem to be a much more heterogeneous population than initially perceived and may differentiate into tissue-specific or tissue-unrelated cell types (2, 4). MSCs are perivascular cells (9, 10), and there is no definitive evidence showing that MSCs have the capacity for asymmetric cell division (11), a characteristic of conventional stem cells (12).

However, there is great confusion in the literature regarding MSCs, which may be defined as mesenchymal stem cells or mesenchymal stromal cells. Then, the International Society for Cellular Therapy (ISCT) established some criteria for correctly identifying these cells and recommended that they be referred to as multipotent mesenchymal stromal cells. However, the acronym MSC is accepted if the authors report the correct definition of the cells used (4, 13).

Some particularities of MSCs’ biology over other stem cell populations make them more suitable for cell-based therapy to treat multiple pathological conditions. For example, they do not involve ethical issues like embryonic stem cells or require genetic manipulation as induced pluripotent stem (iPS) cells (14). MSCs generate progeny by long-term self-renewal, exponentially increasing the number of cells for engraftment after *in vitro* expansion. Moreover, stem cell populations are usually rare tissue components that yield progenitors to linearly and hierarchically differentiate into other cell types. MSCs retain this property (15); however, they can alter the environment through the paracrine secretion of multiple factors, leading to a cascade and proactive network of stem and immune cells differentiation and activation. To date, MSCs lead other stem cell populations to differentiate into a broader range of cells types (cooperative activity). They can also reduce the differentiation of naïve CD4^+^ T cells into Th1 effector cells and promote a shift towards a Th2 immune response (16). When co-cultured with CD8^+^ T cells, MSCs suppressed lymphoid activation by the secretion of prostaglandin E2 (PGE2), indoleamine 2,3-dioxygenase 1 (IDO1), and transforming growth factor (TGF)-β1. Besides, they downregulated the expression of the natural killer group 2, member D (NKG2D) receptor on the T cells (16). MSCs can suppress the proliferation of NK cells (17) and inhibit the expansion of blood invariant natural killer T (iNKT) cells and γδ T lymphocytes, mainly by the secretion of PGE2 (18). LPS- or TNF-activated MSCs mediated M2 macrophage polarization (19) and, when co-cultured with monocytes, induced the secretion of IL-6 and prevented the differentiation into immunogenic antigen-presenting cells (20). MSCs also skew the differentiation of monocytes towards anti-inflammatory IL-10-producing cells (20) and promote monocyte survival and differentiation into CD206^+^ and CD163^+^ type 2 macrophages. These cells also secreted high levels of IL-10 and CCL18. Moreover, it was observed that MSCs directly induced Treg cells by the secretion of TGF-β and indirectly by triggering the secretion of CCL18 by macrophages, which generated more Treg cells (20, 21). MSCs also inhibited the maturation and activation of dendritic cells (DCs) by the JAK1/STAT3 signaling pathway (22). Collectively, these results indicate that MSCs can downmodulate the immune response at multiple levels and through several pathways (23–30).

Although MSCs are usually associated with immuno-depression (31), it seems that MSCs are not constitutively immunosuppressive. They may require a ‘licensing’ step provided by inflammatory molecules like IFN-γ, TNF, or TLR ligands (32) under specific conditions, which can explain some apparently contradictory roles of MSCs in inflammation. Indeed, a pro-inflammatory activity of MSCs may be beneficial in the early phase of inflammation and help build a proper immune response (33, 34). It was published that LPS-stimulated MSCs expressed chemokines receptors and acquired higher mobility. These stimulated cells secreted large amounts of pro-inflammatory cytokines and recruited neutrophils in an IL-8- and migration inhibitory factor (MIF)-dependent manner (35). Although the functional importance of these results remains to be demonstrated *in vivo*, endogenous MSCs may participate in the early phase of pathogen defense (35). Indeed MSCs’ plasticity and adjustable balance between apparent opposite biological functions further support their use in therapeutic trials (33).

Besides the regulation of inflammatory responses, MSCs are important in the control of invading pathogens (33, 36, 37), tissue repair, cell proliferation, apoptosis control, and much more (38, 39). In addition, they are safe to treat lung diseases [reviewed in (40)]. These characteristics prompted several pre-clinical and clinical trials to evaluate their applicability in treating patients with severe COVID-19 and pulmonary post-COVID sequelae, as they may also have anti-fibrotic activity (41). A feature of MSCs is particularly interesting to recover pulmonary structures, as they can reversibly make the mesenchymal-epithelial transition (MET) (42). The MET is triggered by the fibroblast growth factor (FGF) receptor and other growth factors receptors that lead to the upregulation of the transcription repressors Sox2 and Oct4. Then, these molecules suppress the Snail function, a mediator of the epithelial-mesenchymal transition (EMT) (43). Moreover, the transcription factor c-Myc downregulates TGF-b1 and TGF-b receptor 2, and the transcription factor Klf4 activates the epithelial program. These interactions down-flow in the activation of epithelial genes such as E-cadherin, EPCAM, MPZL2, STK17A, CLDN3 (claudin), FAM3C, and many others. Considering that severe COVID-19 leads to a strong inflammatory response in the lungs, broad tissue damage with epithelial compromise, fibrosis, and reduced gas exchange in alveoli, the patients’ recovery can benefit from a multifunction cell population like MSCs.

The COVID-19 was announced as a pandemic in early 2020. Then, several studies indicated that a “cytokine storm” in the lungs is one of the main immunopathogenic mechanisms underlying morbimortality. Moreover, similar to severe acute respiratory syndrome (SARS) induced by avian influenza, COVID-19 patients eventually develop acute respiratory distress syndrome (ARDS). The transplant of MSCs into patients with H7N9 virus-induced ARDS has already been conducted, and it significantly reduced the patients’ mortality compared with control individuals (17.6% against 54.5%, respectively) (44). Among different cell-based therapies, MSCs have a high number of registered clinical trials and possibly more chances to be approved for COVID-19 treatment (45).

In this work, we used single-cell RNA-seq data analysis of MSCs identified in bronchoalveolar lavage (BAL) fluid from mild and severally affected COVID-19 patients, besides control individuals, and observed the high capacity of MSCs to adapt to the environment. When we compared samples from mild cases with samples from control individuals, most genes transcriptionally upregulated after infection were involved in cell proliferation. However, this scenario changed when we compared severely affected with mild COVID-19 cases. In this comparative analysis, MSCs from severe cases upregulated genes involved in cell migration and dispersion in the lungs and induced the γ-activated sequence (GAS) genes, probably triggered by IFNGR1 and IFNGR2. Then, IRF-1 was upregulated, one of the GAS target genes, leading to the interferon-stimulated response (ISR). Besides, they increased multiple genes involved in the MET for tissue repair, virus control, and cell chemotaxis. Regarding cytoplasmic RNA danger sensors, MSCs from severe COVID-19 patients transcribed RIG-1 and MDA5 and upregulated PKR compared with cells from mild cases. In mild and severe cases, the MSCs upregulated genes that code for anti-inflammatory molecules such as IL1RN, AGTRAP, and SOCS1.

In a non-comparative analysis, we observed that MSCs from severe cases did not transcribe many NF-κB upstream molecules, such as Toll-like receptors (TLRs) -3, -7, and 8, tumor necrosis factor receptors (TNFR1 or TNFR2), RANK, CD40, and IL-1R1. Indeed, many NF-κB inhibitors were upregulated, including PPP2CB, OPTN, NFKBIA, and FHL2, suggesting that MSCs do not play a role in the “cytokine storm” observed. Besides, the MSCs from severe cases do not transcribe NLRP3, NLRP6, NOD2, IFN-γ, IFNAR1, IFNAR2, CD80, or CD86. We also evaluated senescence-related gene products, such as NADH dehydrogenase (ubiquinone) ironsulfur protein 6 (Ndufs6), and Erb-B2 receptor tyrosine kinase 4 (ERBB4) and MSCs from severe cases showed signs of senescence. Our results indicate that MSCs adjust their biological response to the pulmonary environment, acting protectively and confirming their applicability in cell-based therapy for COVID-19.

## Materials and Methods

We deployed a processing workflow for Single-cell RNA-seq data analysis in the Santos Dumont (SD) Supercomputer (https://sdumont.lncc.br), which has an installed processing capacity of 5.1 Petaflop/s. It presents a hybrid configuration of computational nodes regarding the available parallel processing architecture. It was necessary due to the large amount of raw data to be processed, over 40 TB.

The COVID-19 datasets of BAL samples of single-cell RNA-seq (scRNA-seq) are available on the Gene Expression Omnibus (GEO) repository (46). Datasets are GSE145926 (47), GSE157344 (48), and GSE167118 (49). Then, three healthy control individuals and three COVID-19 patients with mild symptoms were included in our analysis (47). Regarding severely/critically ill patients, we gathered cells collected from six (47) plus twenty-one (48) individuals in singlicate. Besides samples from nine patients in duplicate (49). These datasets were combined, and we then had forty-five samples from thirty-six severely ill individuals. Only samples that went through the curation and quality control stages were included in this work, justifying the difference in the number of patients per group. The criteria consisted of the availability of descriptive information about the samples, such as a link to supplementary files detailing how the genes’ transcriptional level was measured; access to raw data through the selector SRA link; and all samples in the series had to belong to a single species. Moreover, it was necessary to have the description of the experimental protocol used; have the comorbidities listed and the clinical condition at the time of BAL collection; pass the check if metadata matched the samples’ names; and verification if the scRNA-seq experiments used one of the following protocols: Smart-seq2, Smart-like, Drop-seq, Seq-well, 10xV2 (3 prime and 5 prime), or 10xV3 (3 prime).

According to the authors that uploaded the datasets, the patients were categorized as severe if requiring admission to intensive care unit (ICU) and/or invasive or non-invasive mechanical ventilation. Patients with mild symptoms had fever at the moment of cells collection, respiratory symptoms, and moderate infection with bilateral pneumonia evidenced by computed tomography (CT) imaging. However, they required no admission to ICU or mechanical ventilation. The median age of each group of individuals was 24 years old for the control group, 36 for COVID-19 patients with mild symptoms, and 65 for severely ill individuals.

We used the 10x Genomics pipeline CellRanger v.4.0.0 (50) with default parameters for samples demultiplexing. We aligned the reads and quantified the genes expression using the GRCh38 human genome and a SARS-CoV-2 genome (NC_045512) as reference. We employed Seurat v4.0.3 R package (51) for quality control (QC), Clustering analysis, and differentially expressed genes (DEGs) analysis. We used the following criteria to identify and remove low-quality cells: Unique Molecular Identifier (UMI) count < 301; Genes expressed < 151 and > 3000; and > 20% mitochondrial RNA, as defined in (52). The number of MSCs analyzed, obeying all quality control criteria and phenotypic identification, consisted of approximately 2x10^3^ in control and mild cases and 4x10^3^ in patients with a severe clinical condition.

The Cellranger count software automatically identified the infected cells. Then, in this work, all reads associated with the SARS-CoV-2 received the “sarscov2” prefix, and we executed an R script that created two files: one containing only non-infected cells and the other with infected cells. We selected only SARS-CoV-2 non-infected cells for all analysis to avoid the subversion in gene expression that the intracellular infection could generate. Therefore, we considered that the analysis of uninfected cells present in the pulmonary inflammatory site would provide a more accurate understanding of the MSCs’ function.

We found no MSCs with SARS-CoV-2 intracellular infection in mild cases, and only 32% of the cells from severely infected patients were intracellularly infected. Accordingly, we found no MSCs from mild cases transcribing ACE2 or TMPRSS2 genes, two primary virus receptors for host cell invasion (data not shown). Considering the severe cases, uninfected cells (analyzed in this work) transcribed no detectable levels of both molecules. In contrast, more than 90% of the infected MSCs transcribed high levels of both ACE2 and TMPRSS2 (**Supplemental Materials 1A, B**).

For the clustering analysis, each dataset was normalized and scaled with default parameters. After normalization, we executed the following steps:

The FindVariableGenes function detected the variable genes with the vst selection method and the number of features equal to 2000;
We integrated the datasets with Seurat’s FindIntegrationAnchors and IntegrataData functions by running a canonical correlation analysis (CCA) on each subset;
We performed dimensionality reduction using PCA and UMAP algorithms. For the PCA analysis, we initially included the 30 most significant principal components;
As the final step in the clustering process, we calculated a shared nearest neighbor (SSN) graph between all cells through the FindClusters function with the resolution parameter equal to 0.5. We repeated this analysis for three subsets of data: severe+control, mild+control, and severe+mild.
We selected the cluster corresponding to MSCs in each data subset based on the simultaneous transcription of CD105, CD90, CD73, and no transcription of CD14, CD34, CD45, CD11B, and CD3E genes using Seurat’s FindMarkers and FindConservedMarkers functions (**Supplemental Material 2**). Usually, HLA-DR is part of the panel of molecules not expressed by MSCs. However, as we are analyzing cells from an inflammatory site, we excluded the HLA-DR from the designed phenotype as it may be expressed by IFN-γ-activated MSCs (53).

DEG analysis was performed to identify the cluster of MSCs using the MAST (54) algorithm, with parameters logFC (log fold change) equal to 0.25 and FDR (False Discovery Rate) equal to 0.05, to compare the differentially expressed genes between different subsets. In addition, enrichment analyses were performed with Enrich web-server (55) using gene sets library from the Kyoto Encyclopedia of Genes and Genomes (KEGG) (56) and the Molecular Signatures Database (MSigDB) (57).

To identify the primary biological processes carried out by lung MSCs, we analyzed the genes marked with a positive sign in the column “av_logFC” in the datasheets of COVID-19 patients with mild symptoms versus control individuals (**Supplemental Material 3**) and of severely affected versus mild COVID-19 patients (**Supplemental Material 4**). The positive entries show the genes transcriptionally upregulated in group 1 over group 2. Then, the biological function was assigned to each positive gene, which provided a comparative and general view of the main functions assumed by the MSCs. To build the biochemical pathways, we grouped the upregulated genes by biological function and aligned them in the context of expected cellular responses according to the literature, the KEGG’s databases, and STRING network. For the analysis of mild COVID-19 cases over control individuals, we evaluated 110 genes (**Supplemental Material 3**), and for severe over mild cases, we analyzed 457 genes. Only statistically significant genes, considering the column “p_val_adj” (**Supplemental Materials 3, 4**) were included in the analysis (p ≤ 0.05).

For the non-comparative analysis of MSCs from severe cases, we generated ridgeplots (histograms) and violin plots using Seurat’s *VlnPlot* function. Therefore, in this work, we analyzed transcriptional modulations following two different strategies. First, we performed comparative analyses to gain insight into the gene clusters that were progressively upregulated as COVID-19 worsened, indicating MSCs’ main functionalities at different stages. In this case, the analysis was blind and not directed to genes involved in any particular biological function. We analyzed all transcripts of MSCs from control individuals versus patients with mild symptoms and all positive entries showed the genes transcriptionally upregulated in group 1 over group 2. The same procedure was used to analyze all upregulated genes when comparing severely affected patients over individuals with mild symptoms. This analysis generated two lists containing multiple genes, and we categorized every gene positively indicated according to its biological function. The second analysis strategy was directed to some previously defined genes that mediate specific biological functions under study. This non-comparative strategy was used to analyze molecules involved in antigen presentation and immune regulation, cellular senescence, virus danger recognition and response, and host cell invasion receptors.

## Results

### Profile of MSCs’ Upregulated Genes Comparing Patients With Mild COVID-19 and Control Individuals

**Figure 1:**
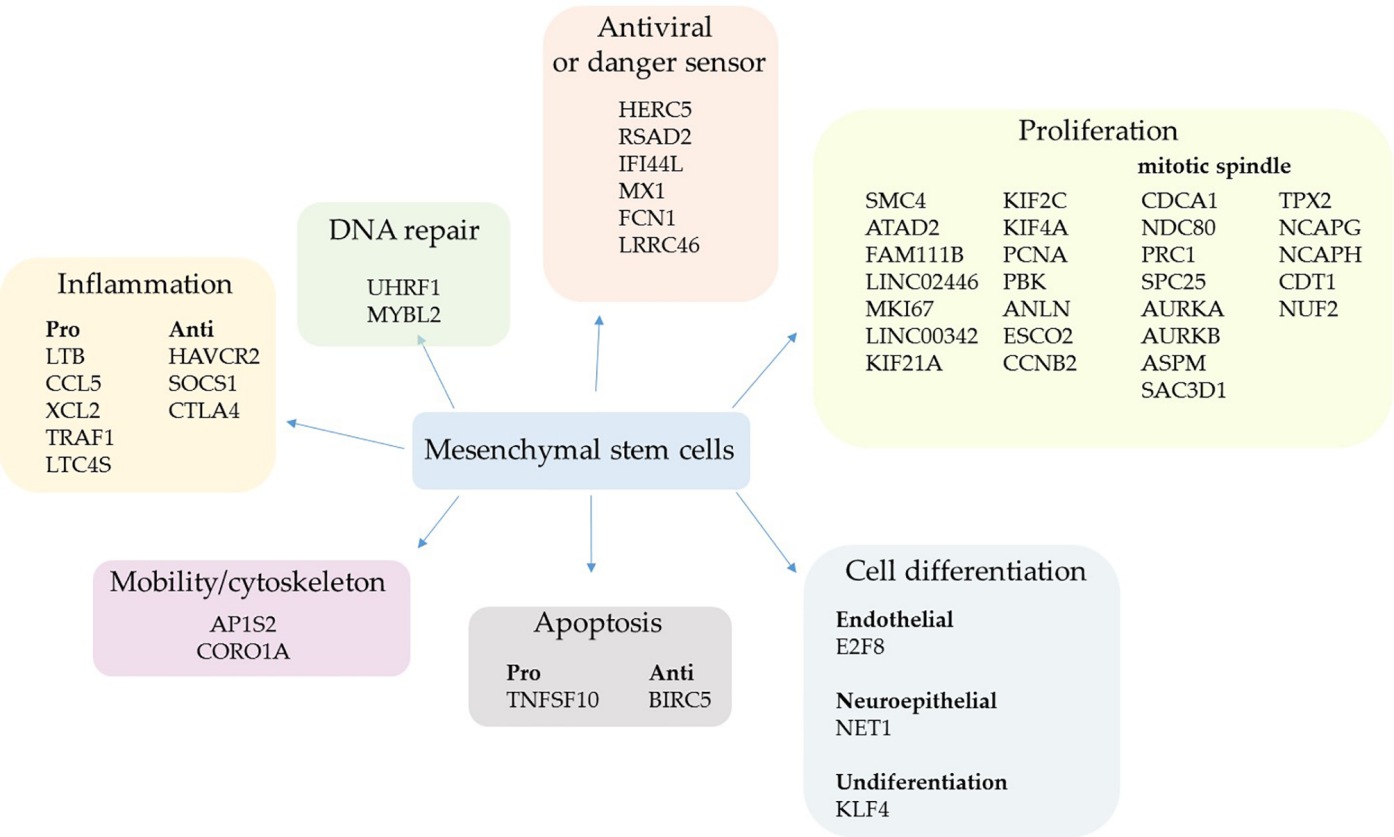
Upregulated genes grouped by biological function in mild cases. The comparative analysis of MSCs’ transcripts from COVID-19 patients with mild symptoms over uninfected control individuals yielded the identification of upregulated genes in sick individuals. The MSCs were analyzed in BAL fluid and the genes were grouped according to their biological function.

The analysis of MSCs from patients with mild COVID-19 compared with control individuals (**Supplemental Material 3**), suggested that these cells were primarily dedicated to proliferation in sick individuals (**Figure 1**). Considering all 110 genes analyzed, almost 1/3 (27%) were related to cell proliferation, and about 19% were related to general metabolism, including mitochondrial function, glucose transport, thymidine and glutamate metabolism, and others (**Supplemental Material 3**).

Two upregulated genes are conventional markers of mitotic cells, *MKI67* and *CCNB2*; both gene products regulate the cell cycle transition at the G2/M stage. The KI-67 protein also maintains the mitotic chromosomes dispersed in the cytoplasm after nuclear envelope disassembly (58). Moreover, some key genes involved in cytokinesis were upregulated, which is the separation of chromosomes and cytoplasm, yielding two daughter cells (59). These genes were *ANLN* (anillin) (60) and some kinesins (*KIF21A*, *KIF2C*, and *KIF4A*) (61) (**Figure 1**). Other upregulated genes control the cell cycle progression, which were *FAM111B*, *PCNA*, *SMC4*, and *ESCO2* (62–65), or multiple cell division checkpoints, as *ATAD2* (66) and *PBK* (67) gene products (**Figure 1**). Although increased DNA repair processes are typically associated with intense cell proliferation, MSCs upregulated only the *UHRF1* (68) and *MYBL2* genes (69) (**Figure 1**).

The second most represented group of genes transcriptionally upregulated was involved in antiviral response and sensing pathogen-associated molecular pattern (PAMPs) molecules (**Figure 1**). The *HERC5* gene product inhibits replication of evolutionarily diverse viruses and boosts the antiviral response (70), the RSAD2 protein (viperin) inhibits the release of viruses from infected cells (71), and IFI44L inhibits virus replication (72). The product of the genes *MX1* (antiviral) and *FCN1* (ficolin 1), an extracellular pattern-recognition receptor (PRR), have already been observed to be upregulated after SARS-CoV-2 infection (73, 74), agreeing with our results.

We observed that the pulmonary environment of mild COVID-19 patients induced the upregulation of only five pro-inflammatory genes in MSCs, which code for the chemokines CCL5 and XCL2, plus TRAF1, which has already been identified as an important inflammatory mediator in the lungs (75). The other upregulated genes were *LTC4S* that codes for the leukotriene C4 synthase, a central enzyme in the metabolism of arachidonic acid (76), and the lymphotoxin-beta (LTB), a soluble inflammatory mediator usually induced by TNF and lymphotoxin (LT)-alpha (77) (**Figure 1**). Regarding anti-inflammatory genes, *HAVCR2* was upregulated; it reduces cytokines, chemokines, prostaglandins, and cell adhesion molecules in the presence of viral infections (78) (**Figure 1**). The SOCS1 gene/protein downregulates pro-inflammatory pathways triggered by TLRs and other membrane receptors at multiple levels (79). Moreover, the cytotoxic T-lymphocyte antigen 4 (CTLA4) molecule was upregulated and it suppresses T lymphocytes activation and function when bound to (CD80) B7.1 or (CD86) B7.2 (80). Although the CTLA4 expression is usually associated with the silencing of T lymphocytes and a subset of B lymphocytes (81), this molecule has already been observed in MSCs inhibiting allogeneic MSCs rejection (82).

Only two gene products that regulate cell differentiation into endothelial (*E2F8*) or neuroepithelial (*NET1*) cells were upregulated (**Figure 1**) besides the *KLF4* gene. This result is interesting because the *KLF4* gene product sustains the self-renewal cycle of stem cells and retains them at an undifferentiated state (83), further supporting the primary assumed function of lung MSCs in mild COVID-19 cases. Other upregulated genes were either pro- or anti-apoptotic and genes that favor cell dispersion (mobility/cytoskeleton) (**Figure 1**).

Some genes associated with other biological functions were individually upregulated in MSCs when comparing mild cases with control individuals. To date, the *DEFB1* gene, which codes the beta-defensin 1, an antimicrobial peptide continuously produced by epithelial cells and other cell types (84) (**Supplemental Material 3**). Moreover, some antioxidants were upregulated, like the product of the *AAED1* gene (**Supplemental Material 3**).

After the comparative analysis of upregulated genes based on their biological functions, we aligned some of the genes/gene products related to cell proliferation and mitotic spindle formation in a sequence of events (**Supplemental Material 5**). As genes associated with the mitotic spindle formation, we included the Aurora kinases A (*AURKA*) and B (*AURKB*). These upregulated enzymes are serine/threonine kinases that associate with the centrosome and the spindle microtubules during mitosis and play an essential role in various cell division checkpoints (85) (**Figure 1** and **Supplemental Material 5**). Other transcripts were upregulated, such as the gene *TPX2*, a spindle assembly factor that intimately interacts with Aurora A and functions in chromosomes segregation (86). Moreover, the *NCAPG* and *NCAPH* genes that code for proteins involved in chromatin condensation (87), and the *SAC3D1* gene that codes for a protein important in centrosome duplication and mitotic progression (88). In addition, the transcription of *PRC1* and *ASPM* genes was upregulated, and they are involved in cytokinesis and the microtubule dynamics at the spindle poles (89). Besides, the *ASPM* gene codes for a protein that seems involved in symmetric stem cells division (90) (**Figure 1** and **Supplemental Material 5**).

Regarding the microtubules’ connection to chromosomes in the mitotic spindle, some components of the Ndc80 complex were upregulated, such as *NUF2* and *APC25* genes. Besides the genes that code for Cdt1, tubulin beta-6 (*TUBB6*), and the kinesins *KIF21A*, *KIF2C*, and *KIF4A* genes (**Figure 1** and **Supplemental Material 5**).

### Profile of MSCs’ Upregulated Genes Comparing Patients With Severe Over Mild COVID-19

**Figure 2:**
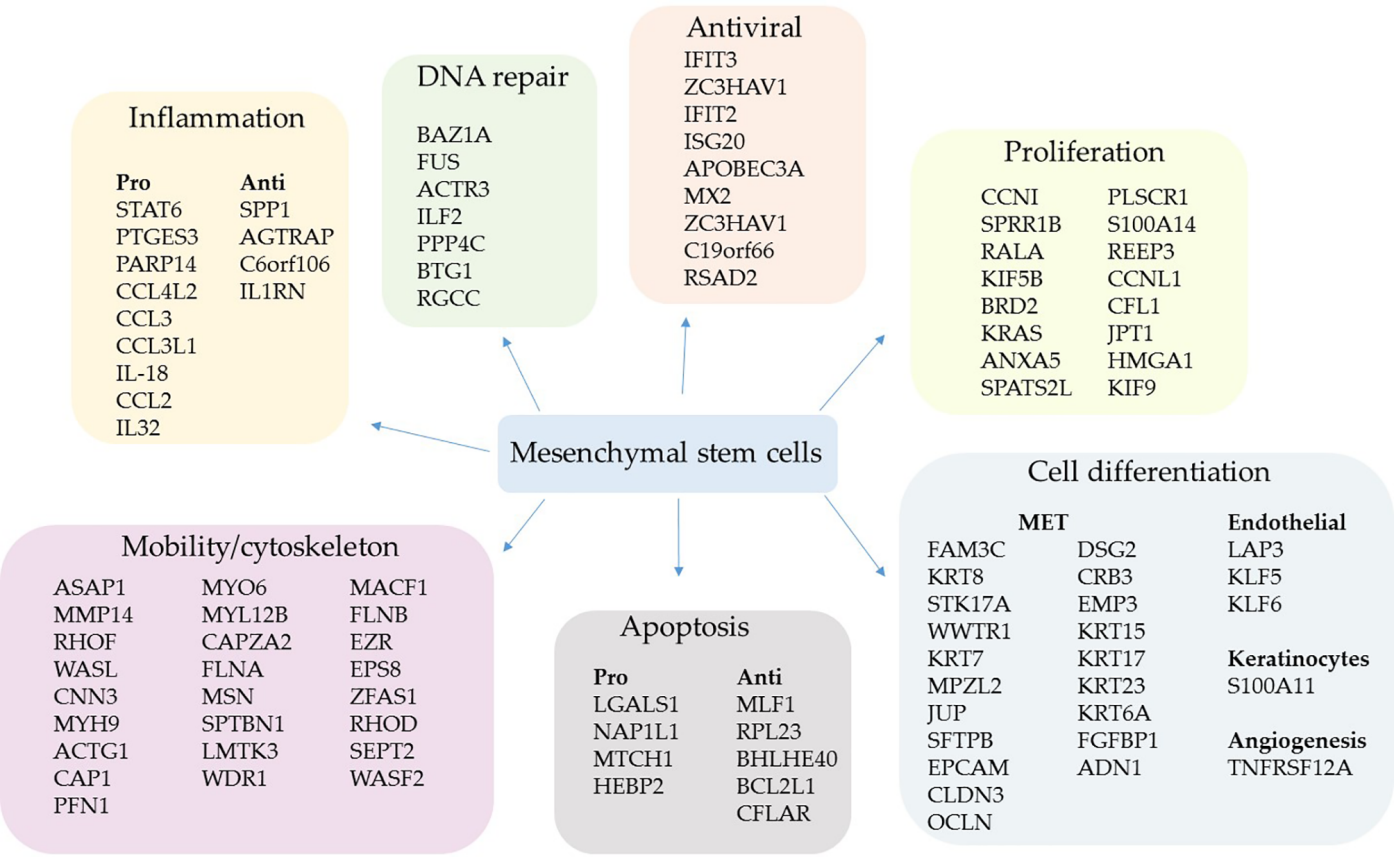
Upregulated genes grouped by biological function in severe cases. The comparative analysis of MSCs’ transcripts from COVID-19 patients with severe over patients with mild symptoms yielded the identification of upregulated genes in critically ill individuals. The MSCs were analyzed in BAL fluid, and the genes were grouped according to their biological function.

**Figure 3:**
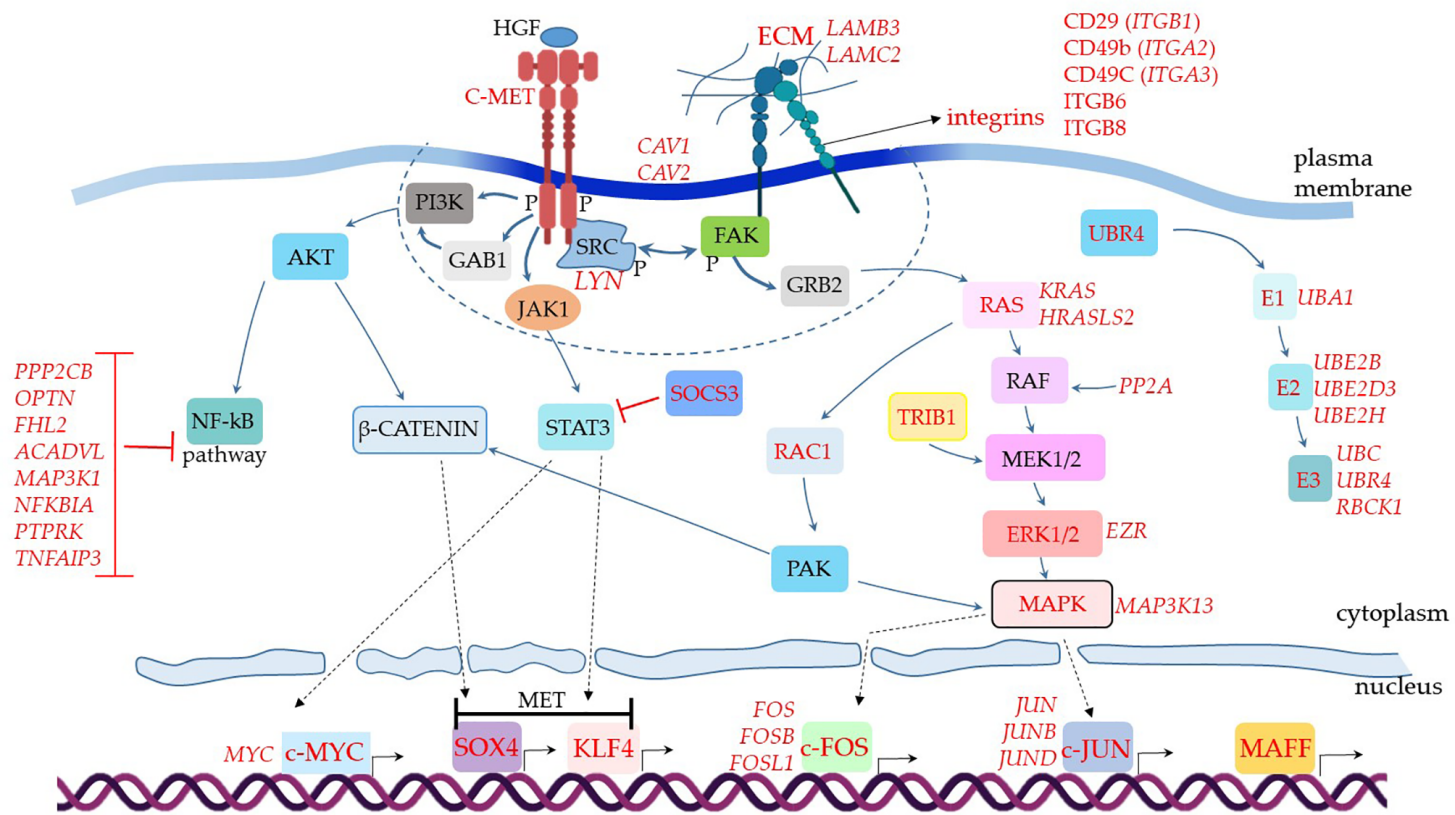
Assumed biochemical molecular pathways triggered in MSCs. The genes were analyzed in MSCs from BAL fluid of COVID-19 patients. This comparative analysis evaluated upregulated genes when comparing COVID-19 patients with severe over individuals with mild symptoms. The upregulated genes are assigned in red, and the genes expressed at a similar level comparing both groups are indicated in black. C-MET means mesenchymal-epithelial transition factor. The dashed line represents membrane-associated and close downstream molecules. Dashed arrows represent molecular physical translocation to the nucleus or activation of nuclear transcription factors.

When we analyzed the genes transcriptionally upregulated in MSCs from severe over mild COVID-19 cases, we observed that multiple biological processes were favored (**Figure 2**), a profile entirely different from that observed in **Figure 1**. Regarding the self-renewal cycle in this comparative condition, the MSCs appeared less committed to clonal expansion in severe cases (**Figure 2**). Instead, many upregulated genes were involved in cell migration in the lungs (**Figure 2**), a fundamental property for any stem cell population. The cell dispersion in stromal tissues is a highly complex process that involves extracellular matrix (ECM) components, ECM receptors, receptor-coupled accessory molecules, and cytoskeleton components that act in a concerted fashion. In this comparative analysis, we observed the upregulation of many cytoskeleton components, including *CNN3* (calponin), *MYH9* (myosin-9), *ACTG1* (actin 1), *PFN* (profilin), *MYO6* (myosin 6), *CAPZA2* (F-actin capping protein), *FLNA* (filamin A), *FLNB* (filamin B), *MSN* (moesin), *CPTBN1* (spectrin beta chain), and *MACF1* (a microtube-actin cross-linker) (**Figure 2**). We also observed the upregulation of two isotypes of laminins, which were *LAMB3* and *LAMC2*, and the integrins *ITGB1* (CD29), *ITGA2* (CD49b), *ITGA3* (CD49c), *ITGB6*, and *ITGB8* (**Figure 3**). These ECM receptors are usually embedded in specialized microregions of the plasma membrane rich in cholesterol and sphingolipids, named lipid rafts. These structures facilitate the lateral mobility of signaling clusters’ components for assembly (91). The endocytosis of rafts may include caveolin-dependent pathways, and we observed the upregulation of caveolin 1 and 2 (*CAV1* and *CAV2*) in MSCs from severe over mild cases (**Figure 3**).

Multiple genes whose products act as cytoskeleton regulatory molecules were also upregulated, such as *RHOF*, *WASL*, *CAP1*, *MYL12B*, *LMTK3*, *WDR*, *RHOD*, *SEPT2*, and *WASF2* (**Figure 2**). Moreover, the gene *EPS8*, which codes for a receptor adaptor protein (92), and the *EZR* gene (**Figure 2**) were upregulated. The *AZR* gene codes for the ezrin, a protein that belongs to the ERM (Ezrin–Radixin–Moesin) family and functions as a cross-linker between the actin cytoskeleton and the plasma membrane. Regarding antiviral components, we found the upregulation of the genes *IFIT3* (93), *ZC3HAV1* (94), *IFIT2* (95), *ISG20* (96), *APOBEC3A* (97), *MX2* (98), *ZC3HAV1* (94), *C19orf66* (99), and *RSAD2* (viperin) (71) (**Figure 2**).

We did not find upregulated IFN-γ transcription in MSCs when comparing patients with mild symptoms over control individuals (**Supplemental Material 3**) or patients with severe over mild cases (**Supplemental Material 4**). This result is significant, as it indicates that MSCs do not secrete one of the main cytokines of the COVID-19 cytokine storm (100). Only the cytokines IL-18 and IL-32 were increased in severe over mild cases and the following chemokines: CCL4L2, CCL3, CCL3L1, and CCL2 (**Figure 2**). Interestingly, some of these chemokines attract immune cells to the inflammatory site and have antiviral activity (101). Besides, the *STAT6* gene was transcriptionally upregulated, a homodimeric transcription factor with many inflammatory and antiviral functions (102), and *PARP14*, whose product acts as a transcriptional co-activator for STAT6 and promotes the activation of a Th2 immune response (103). Finally, the *PTGES3* gene that codes for the prostaglandin E synthase 3 (cytosolic) was upregulated, one of the main inflammatory regulators in inflammatory diseases (**Figure 2**).

MSCs from severe patients also upregulated the transcription of some anti-inflammatory genes, which were *SPP1* (osteopontin) (104), *AGTRAP* (105), *C6orf106* (106), and particularly *IL1RN* (**Figure 2**). This gene codes for an interleukin 1 receptor antagonist, a natural inhibitor of IL-1. In addition to its anti-inflammatory activity, it was described as a potent anti-fibrotic mediator produced by MSCs in the lungs (107).

We also observed the upregulation of many genes involved in the MET or expressed by epithelial lineage-committed cells, such as keratins (*KRT15*, *KRT17*, *KRT23*, and *KRT6A*) (**Figure 2**). The MET is a remarkable function of MSCs (42, 43, 108) and an essential feature in the lungs of patients with severe COVID-19 pneumonia, as long as many epithelial cells die due to the SARS-CoV-2 infection or to secondary inflammatory damage. Besides, MSCs from severe patients upregulated genes involved in angiogenesis (*TNFRSF12A*, also known as TWEAK) (109) plus the endothelial differentiation markers, *LAP3*, *KLF5*, and *KLF6* (110) (**Figure 2**). Moreover, some genes involved in apoptosis induction or resistance were transcriptionally increased (**Figure 2**).

When we analyzed additional genes involved in cellular signal transduction and assembled the puzzle of biochemical signaling pathways of severe cases, we observed a promising scenario for using MSCs as therapeutical elements to treat COVID-19 (**Figure 3**). For example, the transcript of the *MET* gene, which stands for mesenchymal-epithelial transition factor, also known as hepatocyte growth factor (HGF) receptor (HGFR) (111), was upregulated. It codes for the membrane receptor c-MET. We did not observe MSCs transcribing HGF, the soluble ligand of c-MET. However, subpopulations of pulmonary epithelial cells such as secretory, ciliated, and squamous transcribed this mediator, indicating that it is available for MSCs stimulation in patients’ lungs (data not shown).

The c-MET is a tyrosine kinase receptor stimulated by the binding of proteolytically activated HGF, leading to receptor homodimerization and phosphorylation of cytoplasmic tyrosine residues (**Figure 3**). These initial events activate the receptor that recruits multiple signaling effector molecules that include the adaptor proteins growth factor receptor-bound protein 2 (GFRB2), phosphatidylinositol 3-kinase (PI3K), v-src sarcoma (Schmidt-Ruppin A-2) viral oncogene homolog (SRC), and GRB2- associated binding protein 1 (GAB1) [reviewed in (112)] (**Figure 3**, molecules represented within the dashed line). Then, multiple signaling pathways diverge and lead to different and complementary cellular responses.

One of the central observations of this biochemical scenario is the negative regulation of the NF-kB pathway (**Figure 3**), a major pro-inflammatory pathway that leads to the production of TNF, IL-2, IL-1, and many other inflammatory mediators (113). This pathway starts with the c-MET (product of the *MET* gene) directly activating the PI3K (114), or the c-MET leading to GAB1 activation (115) that in turn activates PI3K (**Figure 3**). Regardless of the initial events, PI3K activation leads to AKT activity, an intermediate component of the NF-kB pathway (**Figure 3**). Although the c-MET was upregulated in MSCs from severe over mild cases (**Figure 3**), the NF-kB pathway does not seem to function in critically ill patients. We based this conclusion on the observation that multiple membrane or cytoplasmic receptors that could converge to the activation of the NF-kB pathway, were not upregulated in the comparative analysis of severe over mild cases. This does not necessarily mean that these NF-kB-related genes were not being transcribed, only that they could have similar transcriptional levels when comparing the two groups (below the threshold value of 0.25 in log fold change). To evaluate if these genes were being transcribed in MSCs from severe cases, we used the VlnPlot function, which is a non-comparative analysis and gives the absolute values of specific genes transcriptional level (**Supplemental Material 6**). To date, MSCs from severe cases did not transcribe *JAK2*, *IFNAR1*, *IFNAR2*, and *CD40*, (**Supplemental Material 6**), plus *TLR3*, *TLR7*, *TLR8*, *NAIP* (NLRB), *IL-1R1*, *CIITA* (NLRA), or *RANK* (data not shown). Moreover, we observed that multiple molecules that silence the NF-kB pathway were upregulated in the comparative analysis, which were *PPP2CB*, *OPTN*, *FHL2*, *ACADVL*, *MAP3K1*, *NFKBIA*, *PTPRK*, and *TNFAIP3* (116–118) (**Figure 3**). Therefore, although the c-MET can lead to KF-kB activation, this does not seem to be the case in MSCs from severe COVID-19 patients (**Figure 3**).

Moreover, we evaluated the transcription of RAP1 (*TERF2IP* gene) in a non-comparative analysis. RAP1 is an NF-kB activator, important for pro-inflammatory functions of MSCs (119, 120). We observed that a minor proportion of MSCs from the control group transcribed moderate levels of the *TERF2IP* gene (**Supplemental Material 1C**). Moreover, less than 5% of MSCs from patients with mild symptoms and less than 1% of cells from severely affected individuals transcribed this gene (**Supplemental Material 1C**). The upstream signaling components EPAC1 *RAPGEF3*, *RAPGEF6*, EPAC2 *RAPGEF4*, and *RAPGEF5* were not transcribed in MSCs from mild or severe COVID-19 patients (data not shown).The AKT is a central molecule that triggers other branches of intracellular signaling pathways, including the β-catenin *via*, which is probably active *in vivo* in MSCs comparing our data of severe over mild cases. This scenario is plausible because this pathway can lead to the upregulation of the transcription factor SOX4, as we observed (**Figure 3**) (121). Moreover, SOX4 is critical for MET, an assumed primary biological function of MSCs in severe cases according to the great number of MET genes that were upregulated (**Figure 2**). In parallel, the c-MET-associated kinase JAK1 (**Supplemental Material 6**) can lead to STAT3 activation and upregulation of another MET critical transcription factor that was upregulated, the *KLF4* (122) (**Figure 3**). Both *SOX4* and *KLF4* can induce the transcription of multiple MET genes, and indeed many of these target genes were upregulated in our analysis (**Figure 2**, MET). Alternatively, the KLF4 transcription factor can be activated by STAT-independent pathways, and the upregulated KLF4 (**Figure 3**), KLF5, and KLF6 (**Figure 2**) can play a role in other biological processes besides the differentiation into epithelial cells (123). Alternative pathways that induce KLF4 activation may be functional in MSCs from severe cases. This is possible because we observed the upregulation of *SOCS3*, whose gene product downregulates STAT3 activity (**Figure 3**).

Moreover, the c-MET>JAK1>STAT3 pathway (124) may lead to the activation of another critical transcription factor that was upregulated in our analysis of severe over mild cases, the c-MYC (**Figure 3**). The *MYC* gene can also be upregulated by multiple STAT-independent biochemical pathways (125), and c-MYC activity leads to several biological cell responses (126), including cell adhesion and migration, DNA repair, proliferation, and others.

As we observed the upregulation of multiple genes related to cell dispersion (mobility), we analyzed some molecules that participate in focal adhesion (FA), which are large macromolecular clusters present in specialized plasma membrane regions. The FAs contain integrins and are responsible for intermediating the mechanical force between ECM components to the cytoskeleton. We found several integrins upregulated in MSCs from severe over mild patients (**Figure 3**). After integrin engagement, the focal adhesion kinase (FAK) becomes autophosphorylated and creates a high-affinity binding site for Src kinases, allowing their autophosphorylation. Then, activated Src members further phosphorylate FAK on additional tyrosine residues (127). In our analysis, we observed the upregulation of the Src kinase Lyn (**Figure 3**). This initial interaction forms a signaling platform that triggers the engagement of GRB2 to the pathway, and Ras is recruited in sequence (**Figure 3**). The following signaling cascade includes RAF, MEK1/2, ERK1/2, and MAPK (128), and many of these components were upregulated in our analysis (**Figure 3**). The c-MET receptor can also directly stimulate the Ras component of the *via* (129), an alternative branch not included in **Figure 3**. In addition, the upregulated *TRIB1* gene product can further activate the MEK1/2 response (130) (**Figure 3**).

The RAS component may alternatively lead to the activation of RAC1, which is followed by the activation of PAK (131). At this point, PAK can further stimulate the β-catenin pathway, reinforcing the MET, and/or stimulate MAPK, whose family member *MAPK3K13* was upregulated in MSCs from severe over mild cases (**Figure 3**). One of the outcomes of this pathway is the activation of the transcription factors c-JUN, which had the family members *JUNB* and *JUND* upregulated, and c-FOS with *FOSB* and *FOSL1* genes upregulated (**Figure 3**) (132). In addition, the c-JUN and c-FOS activate the transcription of numerous other genes, including genes that regulate cell migration, survival, proliferation, adhesion to a substrate, and much more. Both c-FOS and c-JUN are members of the Activator Protein 1 (AP-1) that is a generic name for different sets of homo- or heterodimers made up of members of the Fos, Jun, Maf, including *MAFF* (133) (**Figure 3**), and ATF multigene families (134).

Finally, MSCs from severe cases upregulated numerous genes involved in protein ubiquitination (**Figure 3**), a process generally associated with cellular components degradation. However, multiple biological functions have been attributed to the ubiquitin pathway, such as signal transduction, cell cycle regulation, mitophagy, and antiviral activity [reviewed in (135)].

### Danger Recognition in MSCs From Patients With Severe COVID-19

Although we selected only SARS-CoV-2 uninfected MSCs for our analysis, to avoid the profound transcriptional and general biological subversion induced by the intracellular infection, the cells were obtained from a pulmonary inflammatory ambient. Therefore, it was expected that the MSCs analyzed would express a repertoire of PRRs that could recognize viral (danger) PAMPs (136).

**Figure 4:**
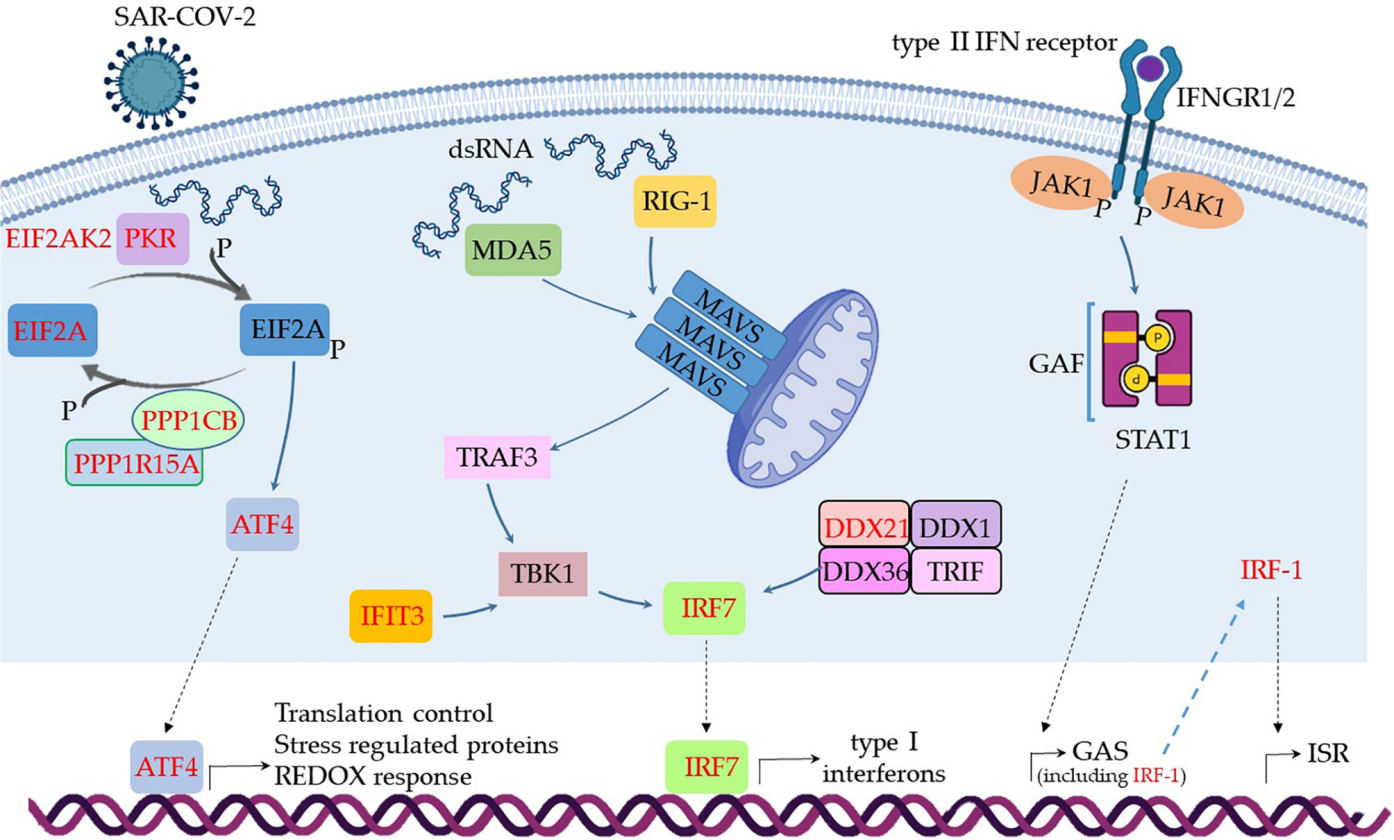
Assumed biochemical pathways involved in virus sensing and antivirus response. The analysis of SARS-CoV-2 DAMPs recognition and antivirus response of MSCs was aligned in a cellular biological condition. The upregulated genes are assigned in red, and the genes expressed at a similar level comparing the group of patients with severe over patients with mild symptoms are indicated in black.

The results shown in **Box 1** indicate that few virus danger sensors were active in MSCs from severe cases, basically RIG-1, MDA5, and PKR, with the associated molecules RIG-G, LGP2, MAVs, TBK1, TRAF3, and IRF7 (**Box 1**). Then, we aligned the transcribed and upregulated molecules involved in danger recognition and antiviral response in MSCs from severally affected COVID-19 patients, and the general scenario is illustrated in **Figure 4**.

Box 1 **Main biological pathways involved in virus sensing and antivirus response.**

**Table:** 

Cytoplasmic RNA sensors		
protein/gene	Severe over control	Severe over mild
TLR3/*TLR3*	NU	NT*
TLR7/*TLR7*	NU	NT*
TLR8/*TLR8*	NU	NT*
NOD2/*NOD2*	NU	NT*
NLRP3/*NLRP3*	NU	NT*
NLRP6/*NLRP6*	NU	NT*
RIG-1/*DDX58*	+1,338	TR*
MDA5/*IFIH1*	+1,525	TR*
RIG-G/*IFIT3*	+2,609	+1,023
CIITA/*CIITA*	NU	NT*
NAIP/*NAIP*	NU	NT*
LGP2/*DHX58*	+0.788	TR*
DHX9/*DHX9*	NU	NT*
DHX15/*DHX15*	NU	NT*
2′-5′-oligoadenylate synthetase/*OAS1*	+1,828	NT*
latent Rnase (RNaseL)/*RNASEL*	NU	NT*
Protein kinase RNA-activated(PKR)/*EIF2AK2*	+0,887	+0,966
**Signal transduction**		
mitochondrial antiviral-signaling protein/*MAVS*	NU	TR*
TANK-binding kinase/*TBK1*	NU	TR*
TRAF3/*TRAF3*	NU	TR*
IRF7/*IRF7*	+2,583	+1,081

The analysis of patients with severe COVID-19 over control individuals was comparative and of severe over patients with mild symptoms was comparative and non-comparative. The genes were analyzed in MSCs from BAL fluid and are identified as: NU (not upregulated or not transcribed); NT (not transcribed); TR (transcribed but not upregulated). The plus sign, indicates the level of upregulation in the comparative condition. * Indicates that a non-comparative analysis was performed.

When MSCs in severely affected patients are exposed to cytoplasmic double-strand (ds) RNA, the PKR-dependent pathway is likely triggered, as many components were upregulated in our analysis (**Figure 4**). This pathway starts with the activation of PKR by autophosphorylation after binding to dsRNA, leading to the phosphorylation of eIF2α, the *EIF2A* gene product. This pathway is usually triggered under cellular stress conditions, leading to protein translation arrest, and it must be transient because its chronic activation is deleterious to the cells. Dephosphorylation of eIF2α is then required to restore protein synthesis after the stress-induced attenuation of translation, and two eIF2α holophosphatases are necessary: phosphoprotein phosphatase regulatory subunit 15A (*PPP1R15A*) (**Figure 4**), only expressed in stressed cells, or phosphoprotein phosphatase regulatory subunit 15B (*PPP1R15B*), which is constitutive (137). The antiviral activity of this pathway is based on at least two important fronts: the translation arrest and inhibition of virus replication and the activation of the pro-inflammatory NF-kB pathway. In this case, the activation of NF-kB is unlikely, as many cytoplasmic inhibitors of this pathway were upregulated in MSCs from severe COVID-19 patients (**Figure 3**). Besides its antiviral activity, this pathway leads to the transcription of target molecules involved in REDOX response, cell survival, and migration by activating the transcription factor ATF4 (**Figure 4**) (138).

Another pathway that seems to be functional in MSCs comparing severe with mild cases is dependent on MDA5 and RIG-1, two known sensors that activate antiviral cellular responses (139) (**Figure 4**). Both molecules converge to the activate mitochondrial antiviral-signaling protein (MAVS) found on the outer membrane of mitochondria (140). Then, TRAF3 is recruited (141), leading to TBK1 activation, which can also be activated by IFIT3 (93), another upregulated gene in this condition (**Figure 4**). Then, TBK1 leads to the activation of the transcription factor IRF7, which induces the production of type I (alpha and beta) interferons (142). Although it has been published that the RIG-I/MDA-5–MAVS signaling pathway is possibly inhibited by the SARS-CoV-2 membrane (M) protein (143), this pathway seems to be active at least in uninfected MSCs (**Figure 4**), as the transcription of IRF7 was upregulated (**Figure 4**). The activation of IRF7 can alternatively be achieved by a complex composed of the helicases DDX1, DDX21, and DHX36 that interacts with the adaptor protein TRIF and cytosolic dsRNA (144). In our analysis, the component *DDX21* was upregulated in MSCs from severe over mild COVID-19 cases (**Figure 4**).

The IFN-γ (a type II IFN) receptors IFNGR1 and IFNGR2 (**Figure 4** and **Supplemental Material 6**) were expressed in MSCs from severe cases, and these receptors signal through JAK1 and JAK2 kinases. However, our non-comparative results of severe COVID-19 patients showed that MSCs are not transcribing JAK2 (**Supplemental Material 6**). Therefore, JAK1 activity would lead to the phosphorylation and homodimerization of STAT1 (**Figure 4**), which is also known as γ-activated factor (GAF) (145). This pathway induces the GAS (γ-activated sequence) response (146) that leads to the transcription of numerous genes that were upregulated in our analysis (147), including the *IRF-1* (**Figure 4**).

Both IRF-1 and type III interferons can induce the interferon-stimulated response (ISR), a robust cellular response important for virus infection control. Type III interferons signal through the IFNLR receptor complex (composed of INFLR1 and IL-10Rβ), and this interaction leads to JAK1 and TYK2 kinases cross-phosphorylation (148). However, we observed that INFLR1, IL-10Rβ, and TYK2 were not transcribed in MSCs from severe cases (data not shown), and therefore this pathway would not lead to ISR. On the other hand, the *IRF-1* gene was upregulated when comparing severe over mild cases, its product is likely activating the ISR. This cellular event is known for leading to the expression of genes such as *ISG20*, *APOBEC*, *IRF7*, *RSAD2* (viperin), *EIF2AK2* (PKR), and many more that were upregulated in our analysis (149). Therefore, our results indicate that type I interferons, that signal through the not transcribed TNFAR1 and TNFAR2 receptors (**Supplemental Material 6**), and type III interferons are not playing a role in MSCs antiviral response in severe cases. This is surprising, as they are some of the most relevant cytokines that compose the first-line defense against viruses. Conversely, and similar to many other viruses, the SARS-CoV-2 has evolved mechanisms for evading the antiviral effects of type I and III IFNs at multiple levels (150). Moreover, the genes *NLRP3*, *NLRP6*, and *NOD2* were not transcribed according to the non-comparative analysis (data not shown). These genes are important PRR sensors for RNA viruses but they do not seem to be employed by MSCs from patients with severe clinical condition.

**Figure 5:**
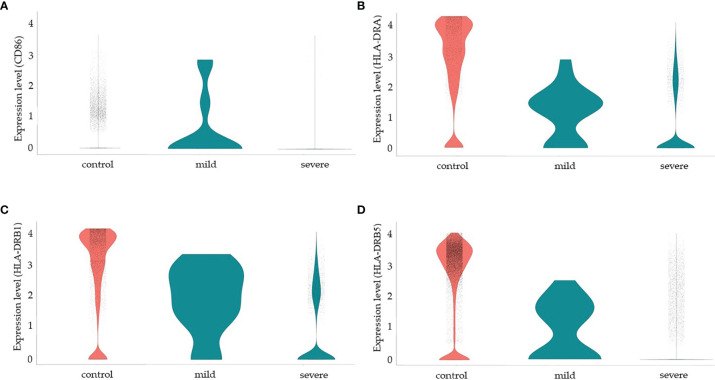
Modulation of co-stimulatory and HLA molecules in MSCs from COVID-19 patients. We made a non-comparative analysis of MSCs from control uninfected individuals and patients with mild or severe COVID-19, as indicated. The molecules analyzed were CD86 **(A)**, HLA-DRA **(B)**, HLA-DRB1) **(C)**, and HLA-DRB5 **(D)**.

Since more severe clinical symptoms are common in older individuals and the median age of this group was 65 years old in our study, we decided to analyze some functional and senescence markers of MSCs. We then analyzed the protein NADH dehydrogenase (ubiquinone) iron-sulfur protein 6 (Ndufs6), a major component of the mitochondrial complex I that mediates MSCs senescence (151). In a non-comparative analysis, we observed that more than 80% of MSCs from the control group transcribed high levels of the *NDUFS6* gene. However, less than 5% of MSCs from the SARS-CoV-2 infected individuals, either with moderate or severe clinical symptoms, transcribed this gene (**Supplemental Material 1D**). Another molecule involved in MSCs senescence is the Erb-B2 receptor tyrosine kinase 4 (*ERBB4* gene). This protein regulates MSCs survival under hypoxia, and *ERBB4* overexpression in aged MSC ameliorates oxidative stress-induced senescence (152). However, we observed no *ERBB4* transcription in either group (data not shown). Serum levels of serotonin have also been implicated in MSCs function/senescence in COVID-19, and serum levels of serotonin and carboxypeptidase A3 (CPA3) (153) have been implicated in COVID-19 severity. However, we observed no transcription of the following serotonin-related genes in MSCs from the three groups of individuals: *HTR1B* and *HTR2B* (serotonin receptors), *PDGFRB*, and *CPA3* (data not shown).Finally, MSCs are considered good candidates for allogeneic transplantation as they express low levels of human leukocyte antigen (HLA) class I (MHC-I) on cell surface and lack the expression of MHC-II and the co-stimulatory molecules CD80, CD86, and CD40 (154). Moreover, MSCs have been demonstrated to be poor stimulators of allogeneic T cell response *in vitro*, which seems to be not due to a deficiency in co-stimulatory signals (155). In humans, there are three MHC-II isotypes, which are HLA-DR, HLA-DP, and HLA-DQ, all encoded by α and β chain genes and we evaluated the transcription of some HLA-DR alleles (**Figure 5**). Different from results previously published by other groups regarding MSCs, and to the best of our knowledge, not obtained from the lungs, our results showed that pulmonary MSCs from control uninfected individuals transcribed the MHC-II α chain (HLD-DRA) and the β chains HLA-DRB1, and HLA-DRB5 (figure 5). However, as we evaluated the transcriptional level, it is possible that those transcripts were not translated or even that the protein is not directed to the cell membrane.

We observed that less than 5% of MSCs from the three groups of patients transcribed CD80 (data not shown) or CD40 (**Supplemental Material 6** and data not shown). Interestingly, MSCs from patients with mild COVID-19 transcribed high levels of *CD86*, *HLD-DRA*, *HLA-DRB1*, and *HLA-DRB5*, suggesting that these cells may play a role in the priming of T lymphocytes and act directly and indirectly in the orchestration of the immune response facing the disease. On the other hand, MSCs from severely affected patients downmodulated the transcription of those molecules substantially. Despite the previous observation that MSCs upregulate co-stimulatory and HLA molecules after exposure to IFN-γ (53), in the pulmonary ambient of severely affected COVID-19 patients, with assumed high levels of this cytokine, this upregulation was not observed.

## Discussion

The WHO declared the COVID-19 pandemic in early 2020, and the world started looking for alternatives for patients’ treatment and management. There is still much to be learned about the disease, but the world is advancing in vaccination campaigns, and health professionals know better how to treat the different COVID-19 manifestations. The main cause of morbimortality is the quick progression of a severe pulmonary inflammatory response, with secondary tissue damage and fibrosis. Another important cause of mortality is the systemic aspect of the disease that yields thromboembolism. Many pharmacological therapeutic alternatives are being developed or repositioned to combat the SARS-CoV-2 infection, besides cell-based alternative therapies. Among these possibilities, pre-clinical and clinical trials using MSCs are among the most promising options, as previous tests in lung diseases indicated that they are effective and safe.

MSCs can linearly differentiate into several cell types that are very important in controlling COVID-19-induced pneumonia and tissue regeneration. Besides, they can modify the pulmonary environment through the paracrine action of secreted soluble factors, many of which were observed to be upregulated in our work. The paracrine activity of MSCs includes the differentiation of other progenitor cells, leading to a proactive cascade of complementary cell types that help in a patient’s recovery.

Among the different approaches to studying the COVID-19 inflammatory response and tissue regeneration, we can highlight the contribution of single-cell RNA-seq data analysis. This approach is a powerful tool that yields the evaluation of thousands of genes in specific cell types that are important to understanding the cellular network that underlies the COVID-19 pathogeny. One of the method’s main advantages is the evaluation of different cell types that integrate the biological network in the pulmonary inflammatory environment *in vivo*. This is a central aspect, which combines single-cell transcriptomics of samples freshly obtained from the patients with no *in vitro* cell culture or other manipulations in laboratory that could alter the cellular biological status. On the other hand, the multiple biochemical pathways and cellular biological responses indicated by transcriptionally upregulated genes cannot be easily confirmed *in vitro*. Therefore, the results predicted by single-cell RNA-seq data analysis remain elusive and prone for confirmation in future essays. In our analyses, we observed that MSCs have high plasticity and adjust their biological functions according to the environment, responding through different sets of transcriptionally upregulated molecules. In the group of patients with moderate clinical conditions over uninfected control individuals, we observed the expansion of a few sets of biologically grouped genes, with the greatest increase in groups of genes related to cell proliferation. Moreover, the second most represented group of genes was related to antiviral activity (156–158). On the other hand, in severe over mild cases, the MSCs response was completely different, as they assumed a genotype compatible with a multipurpose protective cell population. This protection can be illustrated by a refined control of the inflammatory response; moderate transcription of pro-inflammatory molecules, which is important for infection restrain; no significant transcription of main participants of the “cytokine storm” as IFG-γ; and assumed blockage of the NF-kB pathway. Besides, these cells seem to be much more active in antiviral responses and tissue repair, especially leading to epithelial cells differentiation and MET. To the best of our knowledge, this is the first description of MSCs functionally adjusting to the pathogenic ambient, assuming different biological functions.

The most severe cases of COVID-19 are in at least sixty-five years old individuals. In our study, we observed that the patients’ median age of severely affected individuals was considerably higher when compared with the other groups. This observation prompted us to analyze some senescence-related genes of MSCs. To date, several potential mechanisms, including telomere shortening (159), impaired autophagy (160), and especially increased reactive oxygen species (ROS) (161, 162) have been reported to mediate MSCs senescence. Regarding Ndufs6, less than 5% of the MSCs from patients with moderate or severe clinical symptoms transcribed this gene. This downregulation suggests that MSCs in the lungs quickly show signs of senescence, as Ndufs6 is depressed in aged MSCs. However, these cells might be at different stages of senescence and still differentiate into other cell types and play a role in controlling the infection. From our perspective, this possible natural senescence of MSCs in COVID-19 patients further supports the transplantation of MSCs to prevent the worsening of clinical symptoms.

We also evaluated the transcription of RAP1 (*TERF2IP* gene), an upstream NF-kB activator. This pathway ultimately leads to the activation of Raf-1, AF-6, and other transcription factors (reviewed in (163). In agreement with our observation that pulmonary MSCs do not activate the NF-kB pathway in severe cases of COVID-19, we observed that only cells from the control group transcribe high levels of the *TERF2IP* gene.

Serum levels of serotonin have also been implicated in MSCs function in COVID-19, as the treatment of human lung explants with Fluoxerin, an inhibitor of serotonin reuptake, reduced SARS-CoV-2 virus load (164). Besides, serotonin was also implicated in EMT and MET (165). Moreover, serum levels of serotonin and carboxypeptidase A3 (CPA3) (153) have been implicated in COVID-19 severity. However, we observed no transcription of the following serotonin-related genes in MSCs from the three groups of individuals: *HTR1B* and *HTR2B* (serotonin receptors), *PDGFRB*, and *CPA3* (data not shown).Our results show the adaptability of MSCs to the pulmonary environment during the SARS-CoV-2 infection and justify the efforts to establish MSC-based therapies to treat acute COVID-19 and post COVID-19 sequelae.

## Data Availability Statement

All datasets analyzed in this study are available in the GEO repository (https://www.ncbi.nlm.nih.gov/geo/). Accession numbers are GSE145926, GSE157344, and GSE167118.

## Author Contributions

AH-P: analysis of genes lists, biological grouping of upregulated genes, preparation of Figures, and text writing. FS and VS: execution of computational tools and preparation of genes lists (datasheets). DB and SH: manuscript review and editing. All authors have read and agreed to the submitted version of the manuscript.

## Funding

This research was funded by the INOVA program of Fundação Oswaldo Cruz (grant number VPPCB-005-FIO-20-2-34-52).

## Conflict of Interest

The authors declare that the research was conducted in the absence of any commercial or financial relationships that could be construed as a potential conflict of interest.

## Publisher’s Note

All claims expressed in this article are solely those of the authors and do not necessarily represent those of their affiliated organizations, or those of the publisher, the editors and the reviewers. Any product that may be evaluated in this article, or claim that may be made by its manufacturer, is not guaranteed or endorsed by the publisher.
