# Supplementary material for: Pulmonary Mesenchymal Stem Cells in Mild Cases of COVID-19 Are Dedicated to Proliferation; In Severe Cases, They Control Inflammation, Make Cell Dispersion, and Tissue Regeneration
Source: Front Immunol. 2022 Jan 13;12:780900. doi: 10.3389/fimmu.2021.780900 (PMC8793136; doi:10.3389/fimmu.2021.780900)
Supplement: Supplementary file 1 [file DataSheet_1.pdf]

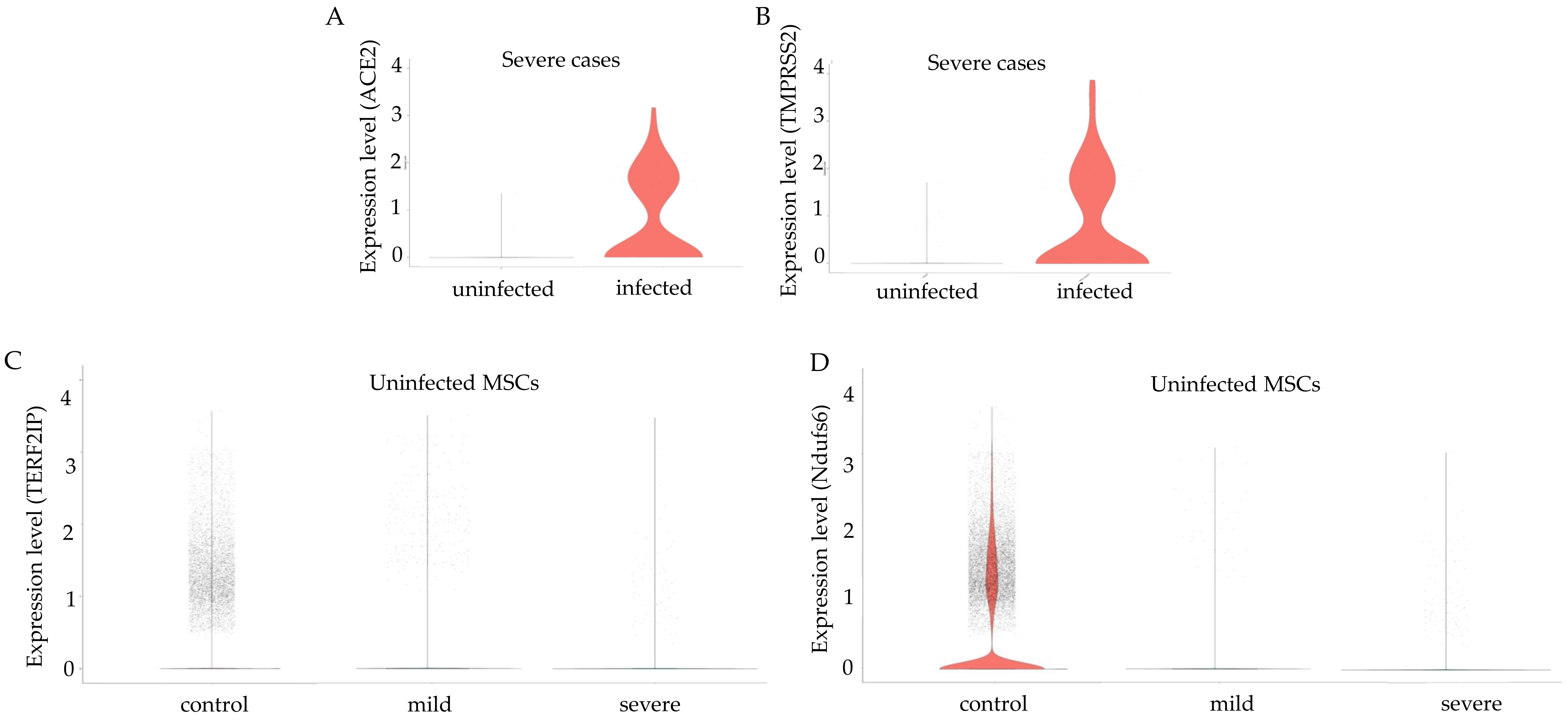

Supplemental material 1: Expression level of molecules involved in MSCs infection, senescence, or biological function. Analysis of *ACE2* (A) and *TMPRSS2* (B), two main SARS-CoV-2 receptors for cell invasion, in MSCs from severe clinical cases. Uninfected and SARS-CoV-2 infected MSCs were analyzed separately. Non-comparative analysis of the genes *NDUFS6* (C) and *TERF2IP* (D) in uninfected MSCs in control individuas and COVID-19 patients with mild or severe clinical symptoms, as indicated.
