# Supplementary material for: Pulmonary Mesenchymal Stem Cells in Mild Cases of COVID-19 Are Dedicated to Proliferation; In Severe Cases, They Control Inflammation, Make Cell Dispersion, and Tissue Regeneration
Source: Front Immunol. 2022 Jan 13;12:780900. doi: 10.3389/fimmu.2021.780900 (PMC8793136; doi:10.3389/fimmu.2021.780900)
Supplement: Supplementary file 2 [file DataSheet_2.pdf]

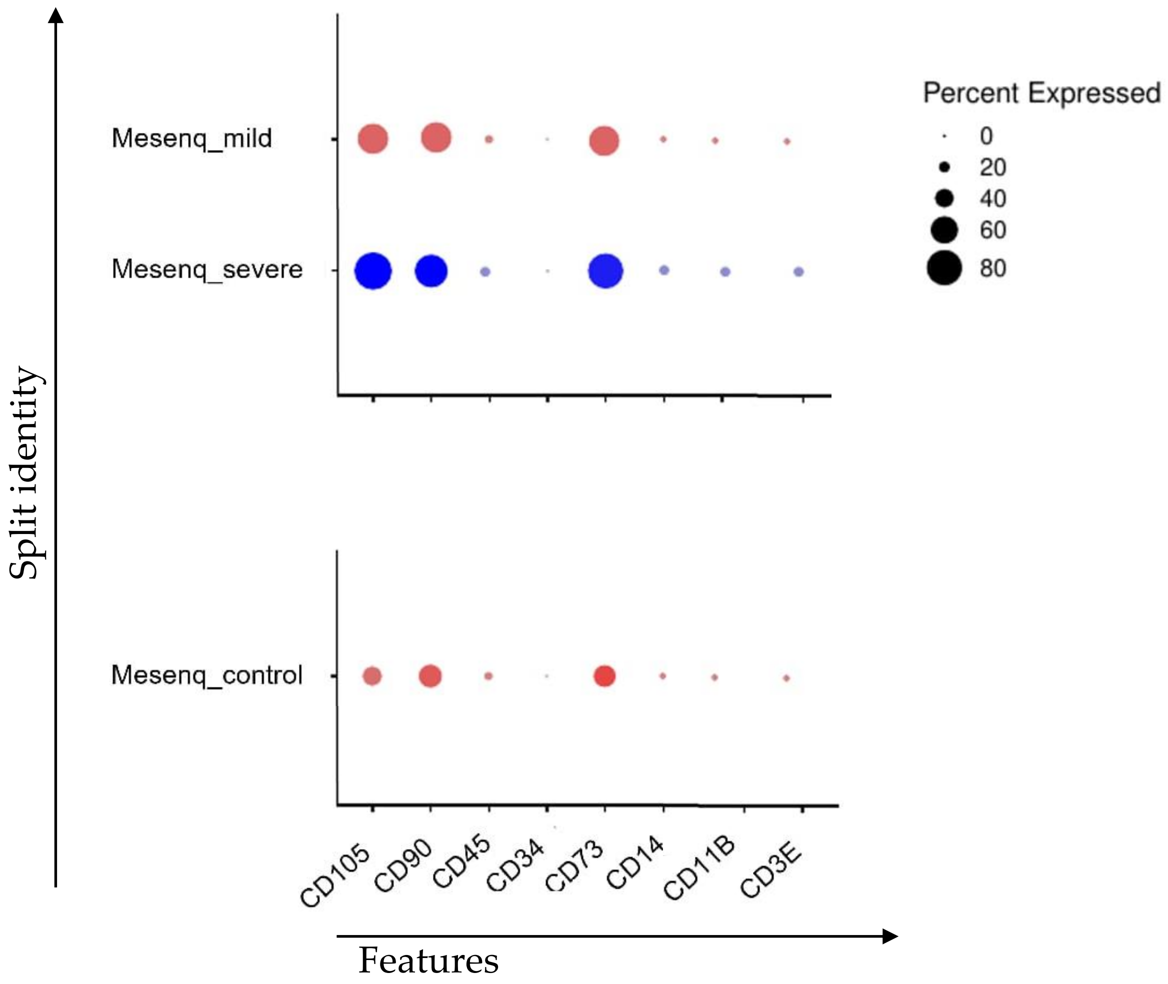

Supplemental material 2: Quality control dot plots of MSCs phenotype. The BAL cells selected for analysis, transcribe the *CD105*, *CD90*, and *CD95* genes. Simultaneously, *CD34*, *CD73*, *CD14*, *CD11B*, and *CD3E* are not transcribed in the clusters corresponding to MSCs for control, mild and severe COVID-19 datasets.
