# Supplementary material for: Pulmonary Mesenchymal Stem Cells in Mild Cases of COVID-19 Are Dedicated to Proliferation; In Severe Cases, They Control Inflammation, Make Cell Dispersion, and Tissue Regeneration
Source: Front Immunol. 2022 Jan 13;12:780900. doi: 10.3389/fimmu.2021.780900 (PMC8793136; doi:10.3389/fimmu.2021.780900)
Supplement: Supplementary file 5 [file DataSheet_5.pdf]

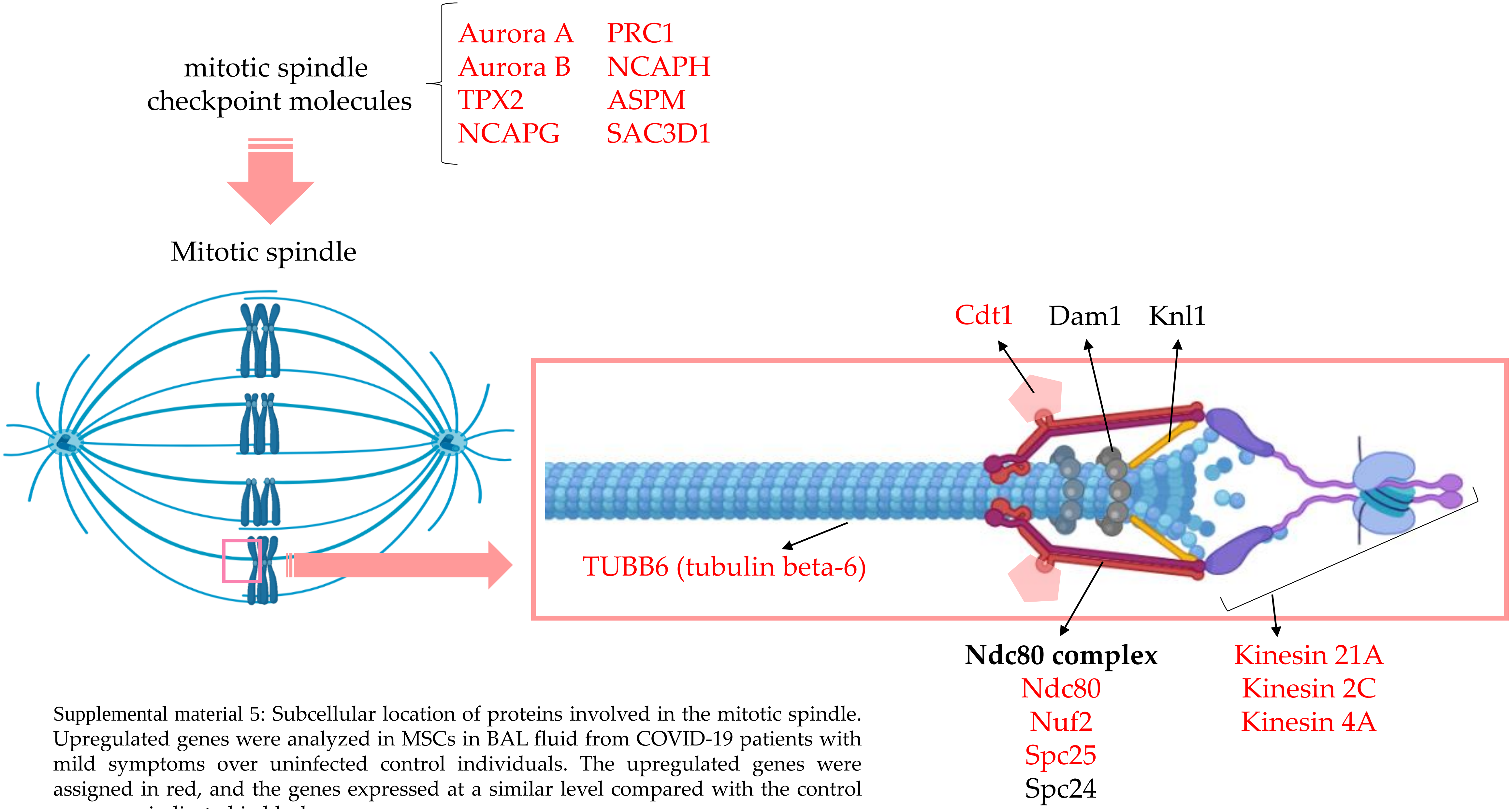

Supplemental material 5: Subcellular location of proteins involved in the mitotic spindle. Upregulated genes were analyzed in MSCs in BAL fluid from COVID-19 patients with mild symptoms over uninfected control individuals. The upregulated genes were assigned in red, and the genes expressed at a similar level compared with the control group are indicated in black.
