# Supplementary material for: Pulmonary Mesenchymal Stem Cells in Mild Cases of COVID-19 Are Dedicated to Proliferation; In Severe Cases, They Control Inflammation, Make Cell Dispersion, and Tissue Regeneration
Source: Front Immunol. 2022 Jan 13;12:780900. doi: 10.3389/fimmu.2021.780900 (PMC8793136; doi:10.3389/fimmu.2021.780900)
Supplement: Supplementary file 6 [file DataSheet_6.pdf]

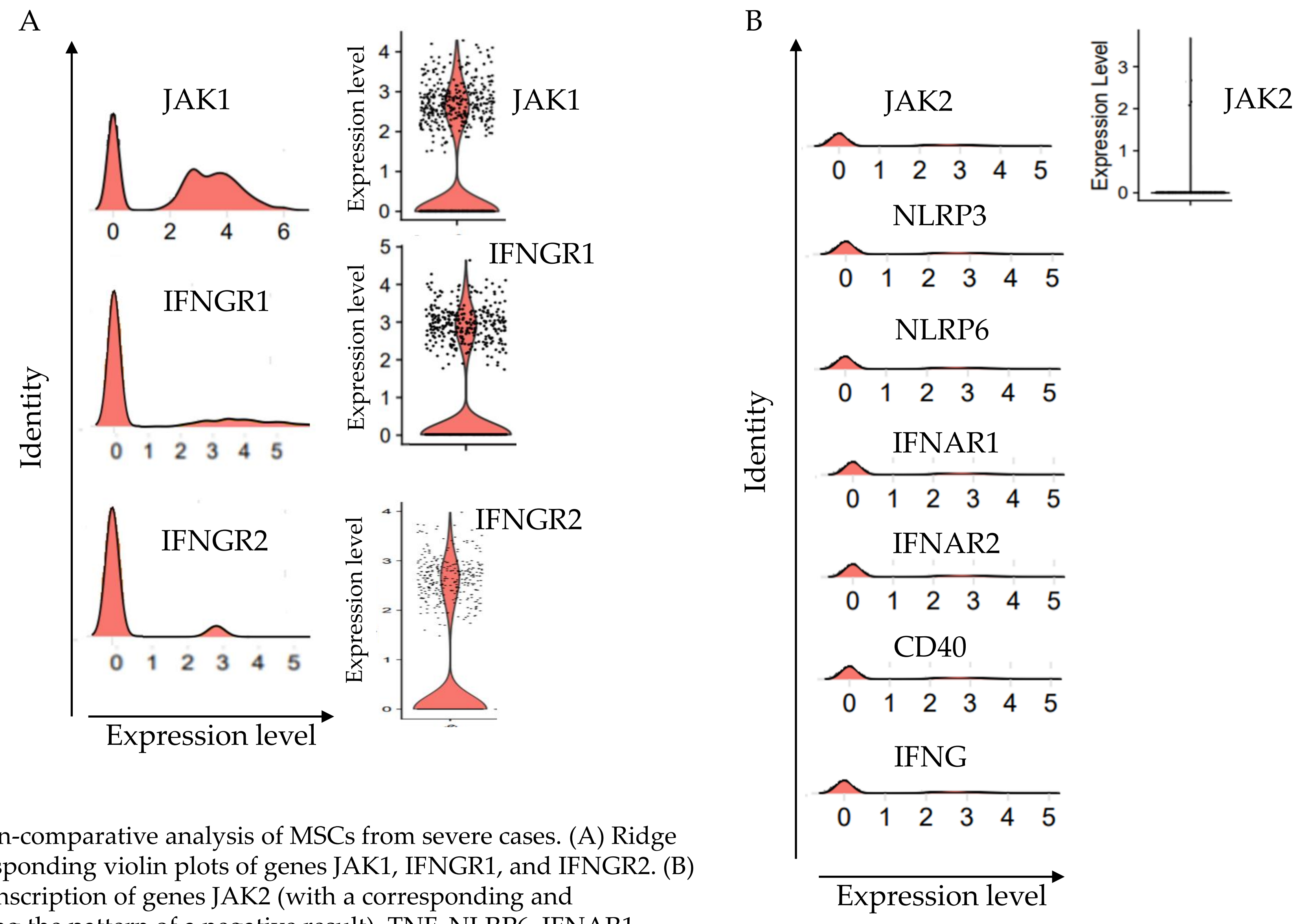

Supplemental material 6: Non-comparative analysis of MSCs from severe cases. (A) Ridge plots (histograms) and corresponding violin plots of genes JAK1, IFNGR1, and IFNGR2. (B) Ridge plots indicating no transcription of genes JAK2 (with a corresponding and illustrative violin plot showing the pattern of a negative result), TNF, NLRP6, IFNAR1, IFNAR2, CD40, IFNG (IFN-γ).
